# T315I^+^ tyrosine-kinase independent CML cells resistance

**DOI:** 10.18632/oncotarget.18573

**Published:** 2017-06-19

**Authors:** Franck Emmanuel Nicolini, Elodie Grockowiak, Véronique Maguer-Satta

**Affiliations:** Department of Hematology, Centre Léon Bérard, INSERM, Centre de Recherche en Cancérologie de Lyon, Centre Léon Bérard, Lyon and University of Lyon, Villeurbanne, France

**Keywords:** chronic myeloid leukemia, tyrosine-kinase inhibitors, resistance, T315I, ER alpha 36

Chen et al. describe in their article [1] a novel BCR-ABL independent TKI-resistance mechanism involving the overexpression of the Estrogen receptor splicing variant α36 (ERα36) in chronic myelogenous leukemia (CML), highlighting its particular implication in ABL wild-type and T315I-mutated engineered and primary cells. Targeting the abnormal activity of the ERα36 in these cells by specific (SNG) inhibitors ±TKI, selectively induce their apoptosis *in vitro* and *in vivo*. Furthermore, they discover that the disruption of the interaction between the BCR-ABL^Tyr177^ and Grb2 by these inhibitors represents the main mechanism of activity in naive and TKI-resistant (T315I^+^) CML cells.

Initial evidence of the restoration of tyrosine kinase (TK) activity in all TKI-resistant CML patients studied came from CL. Sawyers group [2], mainly related to the single amino acid substitution in a threonine residue of the ABL kinase domain (KD) known to form a critical hydrogen bond with Imatinib, the first TKI to be used. They demonstrated that this ABL 944 nucleotide C→T exchange responsible for the T315I KD mutation was not present at diagnosis and thus, appeared on Imatinib, and was responsible for the reactivation of the kinase activity in relapsing and resistant patients. Since then, numerous reports have demonstrated the critical importance of BCR-ABL KD mutations in Imatinib-resistance [3] and have resulted in the mandatory routine assessment (of those) in Imatinib and second generation TKI-resistant patients (Nilotinib, Dasatinib and Bosutinib). In the forest of ABL mutations identified in CML patients, the T315I mutation takes particular importance for four different reasons. Firstly, the majority of reports shows that this mutation is the most frequently identified in patients on Imatinib [3] and has become increasingly problematic over the years. Secondly, this mutation confers resistance to all commercialized TKI as shown in engineered murine cell lines or in biochemical assays [4], with the exception of the recently commercialized TKI, Ponatinib. Thirdly, the T315I mutation can be detected using sensitive molecular techniques (PCR-ASO) in chronic phase CML at diagnosis demonstrating that the disease itself might generate such resistant cells, selectable by TKI that would promote disease progression. Lastly, the T315I mutation might not necessarily induce resistance [5] and can be identified in TKI-sensitive patients (ie. in complete cytogenetic remission) who will never show signs of resistance or even eventually enter an undetectable disease state [5]. These observations imply that another, currently undiscovered, genetic event may be necessary to induce TKI-resistance. However, these sensitive T315^+^ patients are thought to be relatively few in number, and the T315I mutation is generally believed to confer a pan-TKI resistance (with the exception of Ponatinib). The transformation potency of the T315I mutation increases the oncogenicity of CML cells, as shown in animal models [6] where the transfection of murine bone marrow cells with T315I^+^ containing vectors increased the bone marrow growth rate (versus BCR-ABL^wt^), however this enhanced oncogenicity is not conferred by an increase in the TK activity as compared to BCR-ABL^wt^ or other mutated forms. This remarkable observation suggests that other mechanisms occur within the leukemic cell that are able to confer a somewhat specific aggressivity of T315I^+^ cells, such as the activation of the RAS/MAPK as described by Chen et al. [1].

Indeed, before the Ponatinib era, the identification of a T315I mutation in a CML (and Ph^+^ ALL) patient was synonymous with very poor prognosis in all phases [7]. None of these patients could be rescued by second-generation TKI, as expected by *in vitro* observations. Therapeutic options were few, including allogeneic stem cell transplantation in eligible patients, interferon-α, or omacetaxine mepesuccinate the latter retaining some specific activity on the T315I clone through an unidentified mechanism. Nowadays, some patients also fail to respond to the third TKI, Ponatinib, although theoretically active *in vitro* on T315I^+^ clones [4] thus suggesting that alternative pathways for TKI-resistance might be activated such as the MAPK described by Chen et al. [1]. Very recently, a new allosteric inhibitor, ABL001, interacting with a myristoylation site of ABL outside of the KD though regulating the activity of ABL, demonstrated substantial clinical activity in T315I^+^ mutated CML patients underlying the importance of targeting other pathways than that of ABL TK activity itself, in order to sensitize TKI-resistant cells to drugs and obtain disease control.

The involvement of sexual steroids in physiologic and leukemic hematopoietic processes has been described for decades [8] and they have been successfully used in the treatment of hematopoietic disorders such as AML and aplastic anemia. Therefore, there are some known close relationships between normal and pathological hematological processes and the role of sex hormones, has not been widely explored to date at the basic biological level, and particularly in CML, a disease more frequent in males. Here, Chen et al. show for the first time the involvement of the deregulation of the ERα36 in CML progression, never shown to date, and that this pathway is potentially targetable by pre-clinically studied compounds.

## CONCLUSIONS

In their original work, Chen et al. bring to light the activation of alternative BCR-ABL independent pathways responsible for TKI-resistance in CML cells and patients, particularly T315I^+^ ones in which therapeutic options remain challenging. These alternative mechanisms, relying on an overexpression of the ERα36 protein and subsequently on a disruption of the interaction between ABL^Tyr177^ and Grb2 (leading to the activation of the MAPK pathway), remain targetable by SNG inhibitors. However, the clinical safety and activity of such compounds remains to be demonstrated.
